# Mechanobiology of Antimicrobial Resistant *Escherichia coli* and *Listeria innocua*

**DOI:** 10.1371/journal.pone.0149769

**Published:** 2016-02-25

**Authors:** Mehrdad Tajkarimi, Scott H. Harrison, Albert M. Hung, Joseph L. Graves

**Affiliations:** 1 Department of Nanoscience, Joint School for Nanoscience & Nanoengineering, Greensboro, North Carolina, United States of America; 2 Department of Biology, North Carolina A&T State University, Greensboro, North Carolina, United States of America; 3 Department of Nanoengineering, Joint School for Nanoscience & Nanoengineering, North Carolina A&T State University & UNC Greensboro, Greensboro, North Carolina, United States of America; Indian Institute of Science, INDIA

## Abstract

A majority of antibiotic-resistant bacterial infections in the United States are associated with biofilms. Nanoscale biophysical measures are increasingly revealing that adhesive and viscoelastic properties of bacteria play essential roles across multiple stages of biofilm development. Atomic Force Microscopy (AFM) applied to strains with variation in antimicrobial resistance enables new opportunities for investigating the function of adhesive forces (stickiness) in biofilm formation. AFM force spectroscopy analysis of a field strain of *Listeria innocua* and the strain *Escherichia coli* K-12 MG1655 revealed differing adhesive forces between antimicrobial resistant and nonresistant strains. Significant increases in stickiness were found at the nanonewton level for strains of *Listeria innocua* and *Escherichia coli* in association with benzalkonium chloride and silver nanoparticle resistance respectively. This advancement in the usage of AFM provides for a fast and reliable avenue for analyzing antimicrobial resistant cells and the molecular dynamics of biofilm formation as a protective mechanism.

## Introduction

In this study we examine the cell wall properties of antimicrobial resistant strains of *Listeria innocua* (Gram-positive) and *Escherichia coli* (Gram-negative) and their controls. We propose that the cell wall properties of antimicrobial resistant strains of bacteria are different from less resistant strains. This is accomplished by the use of a very fast, accurate and novel mechanobiological method for measuring the “stickiness” of bacterial cell walls using advanced force spectroscopy capabilities of an AFM. Our method allows for fresh bacterial cell cultures to be measured within two hours for their stickiness to the AFM tip in air. These measurements are important as cell wall stickiness can be an important predictor of antimicrobial resistance. Furthermore this study addresses the gap in our understanding of the joint role of single cell and microbial community phenotypes and antimicrobial resistance [1]. The measurement of bacterial cell surfaces also helps to determine how cells interface directly with the environment and with each other. In particular, it has been shown that the surface properties of live bacterial cells are modifiable by antimicrobials and antiseptics [2]. Bacterial adhesion (cell-to-surface adherence), cohesion (cell-to-cell adherence) and viscoelasticity have also been identified as factors promoting cell survival and biofilm formation [3,4]. For Gram-negative bacteria, a major functional determinant in the early stages of biofilm formation is bacterial adhesion based on interactions between the lipopolysaccharide (LPS) in the outer leaflet of the outer membrane forming the first point of contact between the bacterial cell and any surface [5]. For Gram-positive bacteria, peptidoglycan, teichoic acids, pili, and polysaccharides have been measured for stickiness and stiffness using atomic force microscopy (AFM) force spectroscopy [6]. The previous result is an example of how new nanotechnological efforts to evaluate bacterial cell wall properties are guiding research into communal growth and survival of bacteria [7].

AFM is a powerful method for the imaging of live cells under different physiological conditions and for the imaging of real-time dynamic processes such as cell growth, cell division, and effects of drugs and other treatments [8–10]. AFM works by tracing an ultra-sharp probe tip over a sample to generate an image of the surface topology with a vertical height resolution of less than 1 nm. Force spectroscopy is an additional capability of AFM. Force spectroscopy measures the nanoscale mechanical properties of the sample such as flexibility and adhesion. In this method, the probe tip is gently pressed down onto the sample while the resulting strains and forces are recorded to detect forces as small as 10 pN. Differences in the stiffness of the cell surface can thus be measured between different bacteria or even between different areas on a single bacterium and relevant to interactions between bacterial cells, biofilms and surfaces. For example, if resistance to metal nanoparticles is due to the presence of specific ion transport pumps in the cell wall, this may also be manifested by a measurable difference in mechanical properties. In another example, biofilm formation is profoundly influenced by bacterial adhesion to a solid surface. Recently, the physicochemical properties of this process have been extensively studied and involve a range of van der Waals, electrostatic and acid–base interactions [11–13]. These studies suggest that AFM force spectroscopy is overall a versatile nanobiophysical measurement technique capable of measuring adhesion and deflection forces as low as a few piconewtons (1 pN = 10^−12^ N), and is therefore a powerful tool for relating variation in cell wall composition to variation in mechanical properties [14].

It is important to predict the mechanical properties of cell walls because the antimicrobial ability of microorganisms are increased when they attach to surfaces and grow as highly organized multicellular communities. This condition is known as a biofilm [15, 16]. The polysaccharide-rich extracellular substance of a biofilm shields bacteria from harsh physical and chemical factors in their environments, facilitates attachment to biotic and abiotic surfaces, and provides for intra-communal nutrient exchange [17]. A biofilm is made by a firm basal layer and a relatively fragile top layer [18] and is accompanied by a change in electric potential of the surface due to charge transfer between bacteria and the surface [19]. Biofilms are typically a few micrometers or several millimeters thick. The polyanionic extracellular polymeric substances (EPS) of a biofilm are 90 to 97% water and may contain polysaccharides, proteins, phospholipids, teichoic and nucleic acids, and other polymeric substances [20,21].

Biofilm-forming pathogens are negatively impacting both industry and human health and are difficult to control [22–24]. In the food processing industry, mechanical blockages and impedances of heat transfer processes due to biofilms increase the corrosion rate of surfaces, and are an obstacle for efforts at sanitation [17,25–28]. Resistance and persistence of microorganisms to sanitizing chemicals and antimicrobial drugs are potentially due to biofilm-related factors such as nonlethal dosages, strain variation, coexistence of multiple types of bacteria (e.g., *Listeria monocytogenes* surviving in *Pseudomonas* spp. biofilms), and altered single-cell dynamics in the growth stage [21,29,30]. For example, the major pathogen that kills cystic fibrosis patients is *Pseudomonas aeruginosa* whose pathogenesis over time evolves into a hyper-biofilm state in patient lungs [31]. Overall, it has been found that 80% of antibiotic-resistant bacterial infections in the United States are associated with biofilms.

There is therefore an urgent need to evaluate antimicrobial materials in relationship to the evolvabilty of bacterial resistance [32–34]. Two common antimicrobial materials are quaternary ammonium compounds and silver. Benzalkonium chloride (BAC) is one of the quaternary ammonium compounds (QACs) used extensively in food and medical industries as a disinfectant [35,36]. Silver has a long history as an antimicrobial agent, dating back to 1000 BCE. In modern times, nanoparticulate forms of silver, copper, and silica have been successfully used for agricultural applications as both antimicrobial and anti-insecticidal compounds. Nanosilver has become an increasingly common component for food packaging and other materials [37]. Sliver nanoparticles(AgNPs) have been shown to be protective against numerous species of bacteria, including *E*. coli[38].

In this study, we used AFM to measure the cell wall stickiness of both Gram-negative and Gram-positive bacterial strains with evaluated levels of resistance to silver and benzalkonium chloride respectively. This work ultimately aids in the identification of quantifiable measures of single-cell surface properties to provide a mechanistic understanding of biophysical parameters that may be critical for modeling biofilm formation, levels of resistance, and removal.

## Materials and Methods

### Bacterial Growth

This study used *E*. *coli* K-12 MG1655 and a field strain of *L*. *innocua* (labelled as strain 232a-1). The *E*. *coli* strain originated from the American Type Culture Collection (ATCC), and the *L*. *innocua* strain originated from turkey processing plants. Silver nanoparticle-resistant *E*. *coli* MG1655 strain was generated by experimental laboratory evolution according to the procedure described in Graves et al., 2015 [38]. BAC-resistant *L*. *innocua* were obtained from incorporating BAC resistance genes *bcrABC*. These genes have been sequenced and characterized in select strains of *L*. *monocytogenes*, with *bcrABC* shown via sub-cloning and phenotypic complementation to confer resistance to BAC [39]. For *Listeria* samples, we grew the cells overnight (16 h) in Trypticase soy broth (TSB) at 37°C on an orbital shaker (120 rpm). The cells were harvested by centrifugation at 2,300 g for 5 min, and the pellets were washed twice in sterile water. This washing step is necessary for characterization of the bacteria on the glass slide [40]. After the final suspension, 10-fold dilutions were made. The optical density at 600 nm (OD600) was measured, adjusted to McFarland Standard 0.5, and further adjusted as needed in the subsequent experiments. The BAC-exposed samples of bacteria and the nanoparticle-exposed samples of bacteria were washed for 10 minutes at 4°C with deionized water. Silver nanoparticles were provided from nanoComposix (San Diego, CA).

### AFM Imaging and Force Spectroscopy of Bacteria

Glass slides were washed carefully with acetone and then sonicated with 100% ethanol and deionized water for 10 minutes. The glass slides were dried with nitrogen gas and cleaned under oxygen plasma for 3 minutes. Each slide had 10 μL of 0.1% poly-L-lysine dropped onto it and allowed to air dry. Next each slide had 20 μL of washed and diluted overnight bacterial cultures (OD600 of 1.0) deposited onto it and air dried for 20 minutes immediately before AFM imaging. The cells were imaged within two hours after the air drying process. Imaging and force spectroscopy measurements were conducted using a 5600LS AFM (Keysight Technologies) operating in contact mode using silicon nitride cantilever tips. The spring constant of the cantilever tips were measured using thermal calibration of the instrument and applied to calculations of AFM measurements. The Kspring force constant for silicon nitride cantilever tips varied from 0.3–0.6 N/m (Applied Nanostructures, CA). The cell volume and size has been calculated using Gwyddion 2.34 [41].

### Data Analysis

Data analysis was performed with R (R Foundation for Statistical Computing; version 2.12.1 [http://www.r-project.org] [42]. Stickiness ratios and compressive deflection (“stiffness”) properties of cell boundaries were calculated. Calculation formulae, listed below, are applied to AFM force spectroscopy curves, where biomolecules on the cell surface adhere to a tip. As biomolecules lose contact with the tip, they dissociate on the retraction curve, producing a sharp change in force and a distinct “snap-off” event in the curve representing tensile adhesion of the material and a return to a baseline force [43].

Calculation Formulae for Stickiness Ratios and Compressive Deflections of Individual Cells:

    For ***y*-value** conversion, **Force (variable *F*)** is calculated as **Force** [nanonewtons] **=**

    **voltage datum** [volts] **× deflection sensitivity** [nanometers / volts] **×**

    **K spring** [newtons / meter], i.e., ***F* = V × *d***_***s***_ **× *k***_***g***_

    **Baseline (variable *h*):** Compute average of the middle 50% of y values to the right of the minimum y value [nanonewtons]

    **Initial compressive deflection (variable *k*):** slope calculated from coordinates representing the first 33% of ***y*** values (**Force** in nanonewtons) going to maximum to the left above baseline [nanonewtons / meter]

    **Tensile adhesion:** absolute difference between minimal ***y*** value and baseline, i.e., **|min(*y*)-*h*|** [nanonewtons]

    **Maximum force:** absolute difference between maximal ***y*** value and baseline, i.e., **|max(*y*)-*h*|** [nanonewtons]

    **Stickness ratio: Tensile adhesion / Maximum force,** i.e., **|min(*y*)-*h*| / |max(*y*)-*h*|** [unitless]

    **K spring** and **deflection sensitivity** are constants reported from the AFM machine (**variables *k***_***g***_ **and *d***_***s***_) [newton / meter] and [nanometers / volts].

    **Compressive deflection (variable *k***_***s***_**)** is calculated from the following relationship:

    1/*k*_*s*_ = 1/(*k*/10^9^) - 1/*k*_*g*_

    which solves to be

    *k*_*s*_ = 1/(1/(*k*/10^9^)—(1/*k*_*g*_))

    If ***k***_***s***_ >>> ***k***_***g***_, then **1/*k***_***s***_ is effectively zero.

## Results

**Fig 1:**
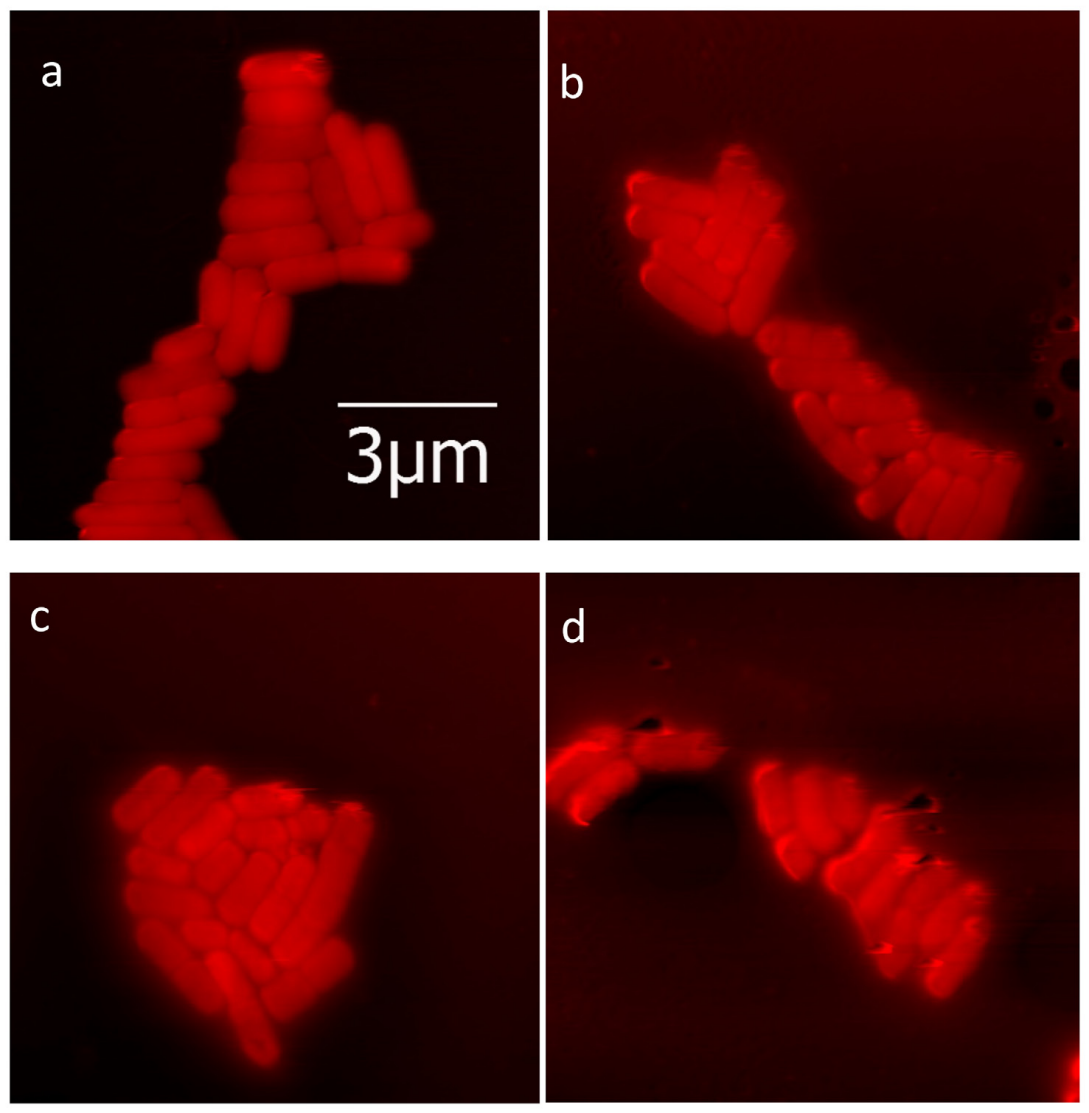
Shaded AFM images of Listeria innocua (a, c) nonresistant and (b, d) BAC-resistant strains (a, b) before and (c, d) after BAC exposure. Scale bar applies to all images.

**Fig 2:**
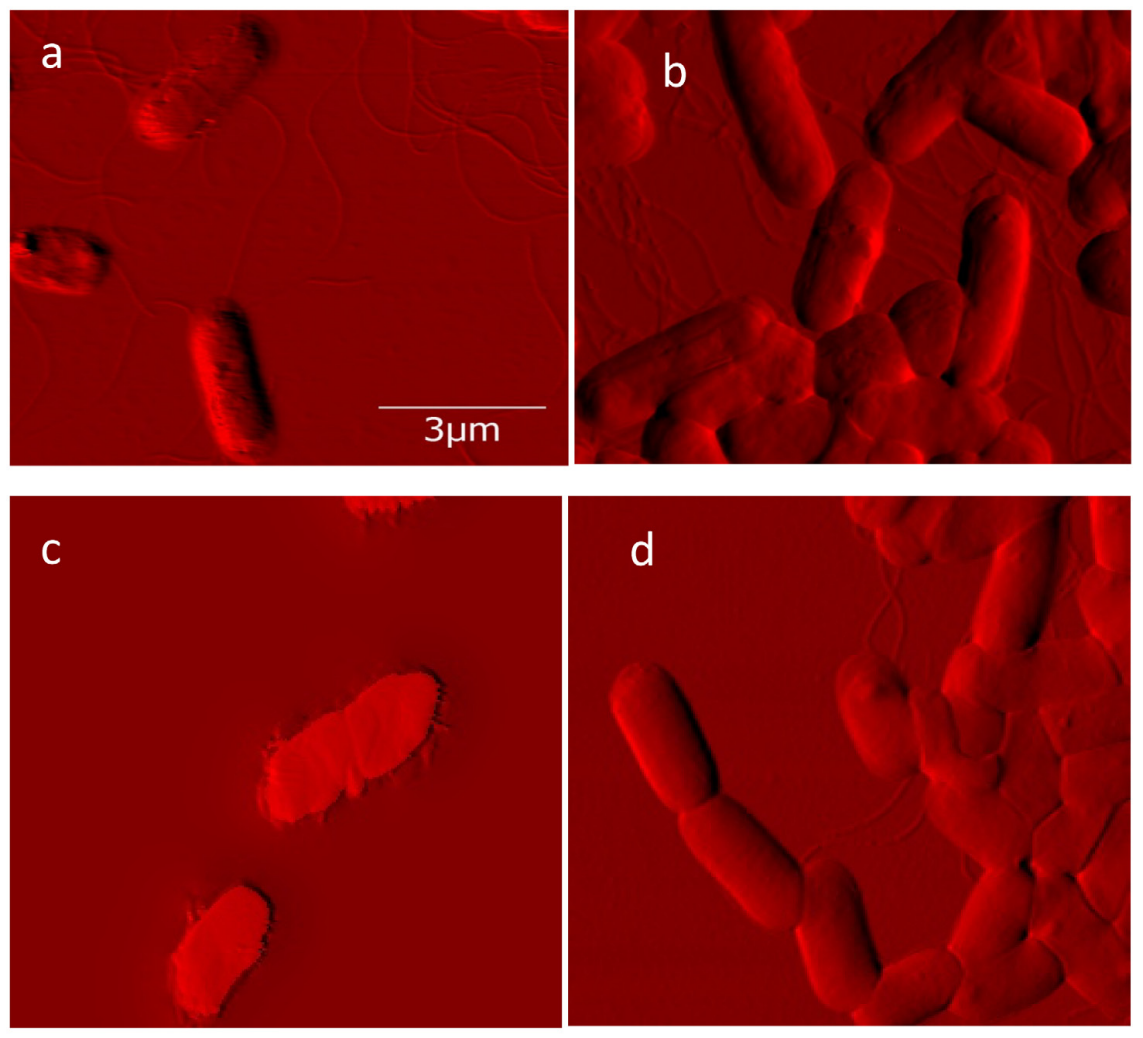
Shaded AFM images of E. coli K-12 MG1655 (a, c) nonresistant (wild-type) and (b, d) AgNP-resistant strains (a, b) before and (c, d) after nanoparticle exposure. Same scale bar applies to all images.

We previously determined adhesive force changes for *E*. *coli* and *Acinetobacteria* spp. cells [44,45]. In this study, we have examined for contexts of biofilm formation and have measured for stickiness. Two bacterial strains were grown in culture medium: Gram-positive *L*. *innocua* field strain (Fig 1) and Gram-negative *E*. *coli* K-12 MG1655 (Fig 2). Cells of *L*. *innocua* and *E*. *coli* K-12 MG1655 were exposed to benzalkonium chloride (BAC) and silver nanoparticles (AgNPs) respectively. Cell surfaces scanned and cell size for each treatment visually observed and then measured through AFM (Figs 1 and 2 and Table 1). According to the Gwyddion software calculations, there to be less change in volume for modified strains during exposure to antibacterials than for the unmodified strains. Cell lengths across factors of antibacterial treatment and genotypes were then measured based on Gwyddion software (Table 1). Length measurements of ten or more different single cells were made for each set of conditions, except for the limited sampling (*n* = 3) for *E*. *coli* wild-type exposed to AgNP. Per each set of conditions, length variation appeared normal, and this was confirmed by the Shapiro-Wilk test for non-normality (for all distributions, *P*>0.05). The quantitative trend of cell length change was consistent with that observed for cell volume, with less change in cell length found for modified strains than for unmodified. Cell length change was not however significant for this modification factor, the antibacterial treatment factor, or the interaction between factors (two-way ANOVA; *P*>0.05). Consistent with how plasticity in phenotype may be a primary outcome to those bacteria adapted to stress, there was a common trend for increased cell length variation in modified strains as induced by antibacterial treatments (AgNPs and BAC respectively for *E*. *coli* and *L*. *innocua*; Table 1), although significance was only found for comparing untreated and treated modified *L*. *innocua* (F test, *P*<0.01) and not modified *E*. *coli* (F test, *P* = 0.12).

**Table 1:** Cell length measurements of resistant and non-resistant strains of *E*. *coli* MG1655 and *L*. *innocua* field strain exposed and non-exposed to AgNP and BAC respectively.

Bacteria name	Number of cell measurements	Resistant	Treated	Mean	Standard Error
*L*. *innocua*	17	-	-	1.82	0.0811
*L*. *innocua*	45	+	-	1.68	0.0530
*L*. *innocua*	11	-	+	1.51	0.106
*L*. *innocua*	16	+	+	1.63	0.155
*E*. *coli*	13	-	-	2.18	0.154
*E*. *coli*	16	+	-	2.24	0.0766
*E*. *coli*	3	-	+	2.80	0.632
*E*. *coli*	10	+	+	2.20	0.153

**Fig 3:**
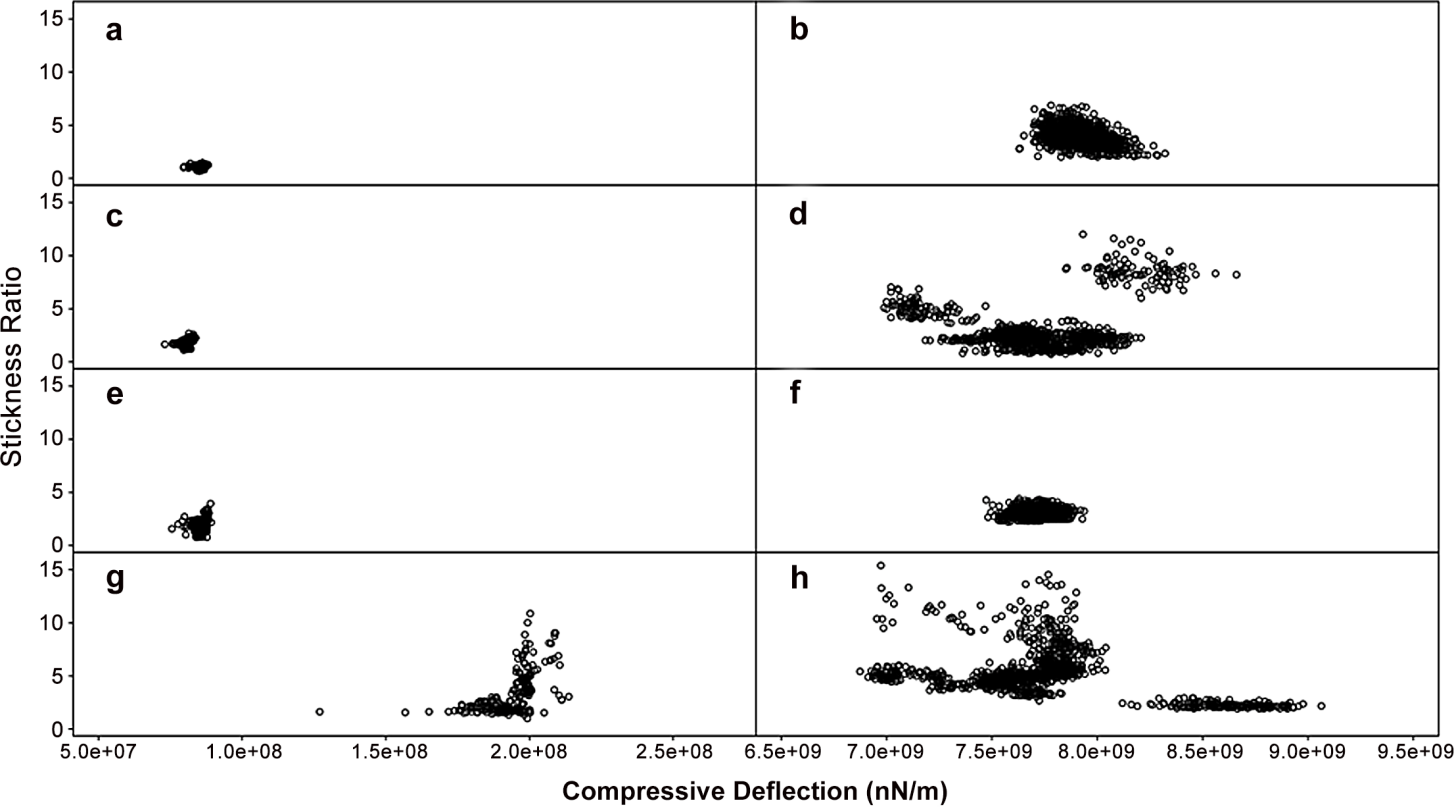
Stickiness ratios and compressive deflections of Escherichia coli K-12 MG1655 and Listeria innocua field strain. (a) E. coli wild-type, unexposed to AgNP. (b) L. innocua unmodified, unexposed to BAC. (c) E. coli wild-type, exposed to AgNP. (d) L. innocua unmodified, exposed to BAC. (e) E. coli resistant, unexposed to AgNP. (f) L. innocua with bcrABC, unexposed to BAC. (g) E. coli resistant, exposed to AgNP. (h) L. innocua with bcrABC, exposed to BAC.

Once the locations of bacterial cells were determined, force curves were obtained at various points on each cell through force cycles of pushing and pulling with the AFM tip. Measures of stickiness ratio and compressive deflection (“stiffness”) properties of cell surfaces are shown in Fig 3. The Gram-positive *L*. *innocua* samples that were tested had proportionally larger force and larger distance components to their linear deflection regions than the Gram-negative bacteria *E*. *coli*. For *L*. *innocua*, we tested 20 bacterial cells per set treatment with 50 replicate AFM measurements for each bacterium. The *L*. *innocua* strain treatments were for a control strain not having the *bcrABC* gene cassette (Figs 1A, 1C, 3B and 3D), and for a modified strain having the *bcrABC* gene cassette (Figs 1B, 1D, 3F and 3H). These treatments were evaluated for the absence or presence of exposure to BAC. For *E*. *coli*, we tested two bacterial cells per treatment with 103 to 174 replicate AFM measurements each. *E*. *coli* strain treatments were for the wild-type K12 MG1655 control strain (not resistant to silver nanoparticles) and, from this wild-type strain, a silver nanoparticle resistant strain was derived through laboratory experimental evolution [12]. Results for the *E*. *coli* wild-type control strain are shown in Figs 2A, 2C, 3A and 3C, and results for the *E*. *coli* AgNP-resistant strain are shown in Figs 2B, 2D, 3E and 3G. Evaluations of these two *E*. *coli* strains were done across contrasting conditions of absence versus presence of exposure to silver nanoparticles (AgNPs).

Statistical distributions and comparisons among the means and variances of compressive deflection and stickiness ratio measures were analyzed. With the exception of *L*. *innocua* with *bcrABC* and no BAC treatment (Fig 3F), distributions of compressive deflections and distributions of stickiness ratios were found to be non-normal (*P*<0.05; Shapiro-Wilk test). Across the two types of bacteria, and as expected for a structurally more rigid Gram-positive cell, *L*. *innocua* had greater compressive deflection than *E*. *coli* for all row-wise comparisons in Fig 3 (*P*<0.05; *t* test and Wilcoxon-Mann-Whitney test). For each type of bacteria analyzed, as may have been due to limitations of sample size combined with variation of cell surface measures, statistically significant differences across arithmetic means were not found for each type of cell surface measure, except for how the silver nanoparticle resistant *E*. *coli* strain exposed to AgNPs (Fig 3G) had a greater compressive deflection than found in other *E*. *coli* treatments (Fig 3A, 3C and 3E) (*P*<0.05; *t* test and Wilcoxon-Mann-Whitney test).

Stress responses in organisms can sometimes be phenotypically plastic, especially where adaptations enable a lineage to vary phenotype in response to a challenging environment. We therefore further investigated for differences in variation across treatment groupings. Significant differences in variation were tested for with the Fligner-Killeen non-parametric test for homoscedasticity with *P*<0.05. When exposed to AgNPs, the evolutionary adaptations of the *E*. *coli* resistant strain associated with a significant difference in variation for both compressive deflection and stickiness measures (Fig 3G) when compared to these measures for the same *E*. *coli* resistant strain without AgNP exposure (Fig 3E), and to the sensitive *E*. *coli* wild-type control strains (Fig 3A and 3C). For the unmodified and artificially modified *Listeria innocua* strains (i.e., without or with *bcrABC*), for both types of cell surface measures, there was significant difference in variation for the strains exposed to BAC versus those strains unexposed to BAC (Fig 3H and 3D versus Fig 3F and 3B respectively). If plasticity of compressive deflection and stickiness is an adaptation, it would appear that the evolved *E*. *coli* strain switches on plasticity in response to stress from AgNPs. *L*. *innocua* appeared to have its own innate plasticity response with or without the *bcrABC* gene cassette.

## Discussion

### Summary of AFM and Approach

Dry AFM force spectroscopy has enabled different types of measurements of cell surfaces including stiffness, elasticity and molecular interactions [46–49]. AFM as a live cell measurement technique has successfully correlated physical properties of the cell surfaces for adhesion forces at precise piconewton and nanonewton levels with production of glycocalyx-containing extracellular polymeric substances (EPS) [50,51]. As cell surface features are associated with biofilm formation we can now study this process at both the molecular and mechanobiology level. We used this approach to pursue novel and rapid measurements of physical adhesion forces for *E*. *coli* and *L*. *innocua* as representatives for Gram-negative and Gram-positive bacteria respectively, and to measure effects of antibacterial materials, AgNP and BAC respectively. We consider the impact of our work to be two fold to have helped further uncover phenotypic plasticity as a mode of bacterial stress response, and to have developed an applied method for rapid analysis of cell surface phenotype.

### Difference in Variation Was Main Effect

In general, it was the difference in variation, and not the difference in mean values of cell surfaces across tested populations of bacterial cells that were found to differ in response to challenges with antibacterial materials. For each strain examined, changes in average mean values were not significant, except for how the silver nanoparticle resistant *E*. *coli* strain exposed to AgNPs had a greater compressive deflection than found in *E*. *coli* evaluated for other conditions of this study (Fig 3). Variation induced by antibacterial treatments for single-cell phenotypes of size, compressive deflection and stickiness was however a common finding for some of the instances examined across both types of bacterial strains in this study. From our visual observations (Figs 1 and 2), some of the variation, especially for cell size, might be due to changes in bacterial growth rate in response to antibiotics. Statistical analysis showed some variation to be significant–for measurements of cell length, compressive deflection and stickiness–and significant variation was most commonly found for comparisons of exposure of antibacterial material to resistant strains. As exhibited by the two strains in this study, phenotypic plasticity may have been a general mechanism of adaptive response to environmental stress, allowing for subpopulations of cells to achieve partial success.

### Phenotypic Differences of the Two Strains

The two bacterial varieties analyzed in this study are very different, and had different phenotypic outcomes to their different modes of treatment. *Listeria innocua* (phylum *Firmicutes*) is a mesophilic Gram-positive soil bacterium, while *Escherichia coli* (phylum *Proteobacteria*) is a thermophilic, Gram-negative gut bacterium. As is consistent with the higher compressive deflection values found in *Listeria innocua* versus *Escherichia coli* for this study, Gram-positive bacteria are stiffer because of a higher turgor pressure than Gram-negative bacteria [52,53]. Both resistant strains’ phenotypes displayed greater stickiness. It is not clear how stickiness influences biofilm formation. For example, one study observed that increased stickiness hindered the reorganization of cells in a biofilm of *Bacillus subtilis* [51]. On the other hand, some studies have shown that increased stickiness was a crucial component of biofilm formation in both *E*. *coli* and *P*. *aeruginosa* [52]. Our single cell study analysis provides the basis for future studies on the formation dynamics of biofilms, including incubation time which is expected to better allow for biofilm formation, and how the influence of stickiness on biofilm formation is dependent on the nature of the substrate [51].

### Genetic Differences

Further work should involve determining those genetic differences that account for how the use of antimicrobial substances selectively alters cell surface properties. A number of genes have been associated with the stickiness phenotype. For example, in *E*. *coli*, the outer membrane protein A (OmpA) protein influences biofilm formation differently on hydrophobic and hydrophilic surfaces via the reduction of cellulose production (cellulose is hydrophilic). OmpA increased biofilm formation on polystyrene, polypropylene, and polyvinyl surfaces (hydrophobic) and decreased biofilm formation on glass (hydrophilic). That study revealed that OmpA induced the CpxRA two-component signal transduction pathway which responds to membrane stress. In *Pseudomonas aeruginosa*, biofilms isolated from the lungs of cystic fibrosis patients show a mucoid phenotype that overproduces alginate. This phenotype is produced by a mutation in the RNA polymerase N (*rpoN*) gene. These mutants are stickier than the wild-type strains[38]. For this study, a number of observations taken together suggest that it is possible that increased stickiness of our silver nanoparticle resistant *E*. *coli* populations may play an important role in their ability to persist in the silver nanoparticle environment [52]. The *E*. *coli* strains used in this study were produced by experimental evolution and their genomes were characterized in Graves et al. 2015 [38]. They differ from non-resistant, wild-type *E*. *coli* K12 MG1655 primarily by single nucleotide polymorphisms in three genes: *cusS*, a sensory histidine kinase in two component regulatory system with *cusR* that senses both copper and silver ions; *purL*, phosphoribosylforml-glycineamide synthetase that is involved in purine synthesis; *rpoB*, RNA polymerase B; and structural variants in *ompR*, outer membrane protein R. At present, we do not know which of these variants contribute to the greater stickiness of our silver nanoparticle resistant strain. However, genetic variants in both *purL* and *ompR* have been associated with biofilm formation in *Photorhabdus temperata* (a bacterium that inhabits the gut of nematodes) and *E*. *coli* respectively [53,54]. The full set of mutations may likely influence levels of transcription for multiple proteins impacting stickiness in silver-containing environments. In addition, a mutation in the intergenic region between *yfdX* and *ypdI* was also identified in the silver resistant strains (frequency = 0.152). This region is of some interest to biofilm formation, as *ypdl* is a putative lipoprotein involved in colanic acid biosynthesis. Anionic colanic acid has been shown to play an important role in biofilm formation in *E*. *coli* [55]. The BAC resistance in the *Listeria innocua* strain used in this study was produced by the introduction of a gene cassette *bcrABC* introduced from *Listeria monocytogenes* H7858, via the pLM80 plasmid. The *bcrABC* gene cassette has been shown to confer BAC resistance in a large number of strains within that species [56]. As the bacteria measured in this study were not sequenced, we do not know if they harbored any additional mutations other than those predicted by the *bcrABC* gene cassette. The pLM80 plasmid consists of at least 69,352 bp (GI: 47018986) and at least 80 genes. Although providing for BAC resistance, the *bcrABC* gene did not appear required for the induced variation of cell surface phenotype.

### Innovation

As an innovative technique, our proposed stickiness measurement method is the first applied method at a cellular level that could be utilized for rapid and accurate study of biofilm-related adhesion. Analysis of bacterial adhesion to a solid surface usually proceeds by radioactive labeling, fluorescence tagging (measured by microscopy or fluorescence), staining of bacteria (with crystal violet and DAPI), Microbial Adhesion to Solvents (MATS) and Contact Angle Measurements (CAM) computed through the equation of Van Oss [23,10], and other methods such as optical force measurement [57]. Various limitations have been found for these methods, for example, laborious enumeration and possible observer errors by microscopy after staining. Furthermore, enumeration by microscopy cannot be used when adhesion of bacteria is studied in a mixed population. Radiolabels are regarded as undesirable due to safety and cost concerns [23]. CAM and MATS methods have not been found to work consistently with respect to each other across different strains of bacteria for measurement of electron donor and electron acceptor properties during adhesion analysis [10].

### Note on Air Versus Liquid

Although liquid-based AFM studies may be an essential next step to see whether or not an air drying method needs to be replaced or utilized to complement the expected advantages of liquid-based AFM, there are several expected advantages for imaging in air and not liquid. These include higher resolution images that more effectively distinguish bacterial structures such as pili and flagella [13,58], and the prevented attachment of suspended particulates and bacterial cells to the tip [13]. The disadvantages of AFM imaging in liquid include fluctuation of solvation forces that will cause lower resolution of the image and force curve [13]. In liquid-based imaging, the surfaces appear smooth and softened, losing resolution of ridges, bumps, or distinct features [52]. Although there have been reports of possible dehydration and other effects to the cell with air AFM [13], measurements of cells remain reproducible [59]. Other concerns about using AFM in dry air relate to water condensation layers, capillary forces and contaminations from both probe and sample that could cause a meniscus pulling the two together [13,14,46]. However, under conditions of 50 to 60% relative humidity, there is no indication of capillary forces [13]. As a preventive protocol, water condensation layers have been avoided in this study by using 1 minute of nitrogen gas spray on sample surfaces before scanning.

### Qualitative Note

From visual examination, there were subtle differences of an uninterrupted surface convexity for resistant strains (Fig 3C and 3D; Fig 2B and 2D) when compared to the more mottled surfaces of sensitive strains (Fig 3A and 3B; Fig 2A and 2C). It is possible that AFM imaging in air drying may conserve this convex versus mottled difference in texture. One of the most striking differences is found for Fig 2C (*E*. *coli*, sensitive and exposed to BAC) where visual examination suggests the increase in mottled surface convexity, reduction of the bacillus morphology, and diminished cell clustering. All three of these observations suggest bacteria in a state of poor physiological health. A qualitative discovery-mode of visual inspection of microscopic imaging remains therefore critical to study further effects of environmental stressors upon different strains of bacteria, and to guide further improvements in quantitative analysis.
